# The Roles of Gut Microbiota Metabolites in the Occurrence and Development of Colorectal Cancer: Multiple Insights for Potential Clinical Applications

**DOI:** 10.1016/j.gastha.2024.05.012

**Published:** 2024-06-07

**Authors:** Wenyue Cheng, Fan Li, Rongcun Yang

**Affiliations:** 1 Translational Medicine Institute, Affiliated Tianjin Union Medical Center of Nankai University, Nankai University, Tianjin, China; 2 Department of Immunology, Nankai University School of Medicine, Nankai University, Tianjin, China; 3 State Key Laboratory of Medicinal Chemical Biology, Nankai University, Tianjin, China

**Keywords:** Colorectal Cancer, Gut Microbiota, Immune Cells, Metastasis

## Abstract

Colorectal cancer (CRC) is one of the most common cancers worldwide. The occurrence and development of CRC are related to multiple risk factors such as gut microbiota. Indeed, gut microbiota plays an important role in the different phases of colorectal cancers (CRCs) from oncogenesis to metastasis. Some specific bacteria such as *Fusobacterium nucleatum (F. nucleatum)* associated with CRCs have been found. However, recently identified bile acid and tryptophan metabolites as well as short chain fatty acids (SCFAs), which are derived from gut microbiota, can also exert effects on the CRCs such as that SCFAs directly inhibit CRC growth. Importantly these metabolites also modulate immune responses to affect CRCs. They not only act as tumor inhibiting factor(s) but also promotor(s) in the occurrence, development, and metastasis of CRCs. While gut microbiota metabolites (GMMs) inhibit immunity against CRCs, some of them also improve immune responses to CRCs. Notably, GMMs also potentially affect the shaping of immune-privileged metastatic niches through direct roles or immune cells such as macrophages and myeloid-derived suppressive cells. These findings offer new insights for clinical application of gut microbiota in precise and personalized treatments of CRCs. Here, we will mainly discuss direct and indirect (via immune cells) effects of GMMs, especially SCFAs, bile acid and tryptophan metabolites on the occurrence, development and metastasis of CRCs.

## Introduction

Colorectal cancer (CRC) is one of the most common cancers and accounts for approximately 10% of all annually diagnosed cancers.^1^^,^^2^ The occurrence and development of CRC are related to multiple risk factors such as gut microbiota. Indeed, gut microbiota not only plays a critical role in normal gut physiological functions such as digestion, biosynthesis of vitamins, generation of heat, gut immunity, and maintenance of gut homeostasis, but also contributes to a wide range of diseases such as tumor.

The roles of gut microbiota in the occurrence and development of CRCs have been widely reviewed. Multiple factors of gut microbiota such as gut dysbiosis, specific pathogenic microbes, metabolites and virulence factors can contribute to the initiation and progression of CRCs. Although there exist debates surrounding the causal relationship between microbial dysbiosis, which is compositional and functional alterations of the gut microbiome,^3^ and colorectal tumorigenesis,^4^ a number of studies have shown that the dysbiosis of gut microbiota is related to CRC development and progression. The characteristic changes in gut microbiota can happen in various CRC stages, including early and advanced CRCs in CRC patients. Recent metagenomic and metabolomic analyses showed distinct gut microbiome-derived phenotypes in early onset of CRCs.^5^ There were enriched *Flavonifractor plauti* and increased tryptophan (Trp), bile acid (BA), and choline metabolism, whereas in late-onset CRC, *Fusobacterium nucleatum* enrichment, short-chain fatty acid depletion, reduced microbial gamma-aminobutyric acid biosynthesis and a shift in acetate/acetaldehyde metabolism towards acetyl-coenzyme A production were found^5^ Indeed, specific bacteria associated with the onset and progression of CRCs have been found, such as *Fusobacterium nucleatum (F. nucleatum)*, *Enterococcus faecalis (E. faecalis)*, *Streptococcus gallolyticus (S. gallolyticus)*, *Bacteroides fragilis* (*B. fragilis*), and Escherichia coli *(E. coli)*. The metabolites from gut microbiota can not only cause initial inflammation but also play an important role in the CRCs from oncogenesis to metastasis. Mechanistically, CRCs can be derived from the metabolites mediated genetic and epigenetic pathways such as histone modifications, DNA methylation, and noncoding RNAs.^6^ However, gut microbiota can also produce a wide range of anticancer metabolites, including bacteriocins, antibiotics, peptides, enzymes, and toxins to inhibit tumor growth.

Notably, recent studies have shown that gut microbiota metabolites (GMMs) such as short chain fatty acids (SCFAs), BA, and Trp metabolites not only directly influence tumor growth^7^ but also indirectly affect genetic and epigenetic regulation and the metabolism in the immune cells, including myeloid-derived immune cells such as macrophages (Macs) and myeloid-derived suppressive cells (MDSCs), and lymphoid-derived immune cells such as regulatory T cells (Tregs) and regulatory B cells (Bregs). These immune cells can express the receptors of SCFAs, Trp, and BA metabolites. ^8^ Their activation may promote the differentiation and alter the functions of immune cells to suppress immune responses to maintain the homeostasis of gut tissues. Notably, gut microbiota mediated immune cells such as immunosuppressive Macs and MDSCs can also assist metastatic dissemination by creating niches which allow tumor colonization. ^9^ However, some GMMs such as SCFAs, BA, and Trp metabolites can also promote antitumor immune responses. Here, we will mainly discuss the effects of recently identified BA and Trp metabolites as well as SCFAs from gut microbiota on the CRCs or CRC-associated immune cells. These GMMs not only directly inhibit tumor growth but also indirectly promote development of CRCs. Especially, they not only suppress immune responses but also promote immunity against CRCs.

## Gut Microbiota and Its Metabolites

SCFAs, BA, and Trp metabolites from gut microbiota not only directly affect CRCs but also indirectly regulate gut and systemic immune response to influence the occurrence, development and metastasis of CRCs.

### SCFAs

SCFAs, mainly including acetate, propionate and butyrate, are from dietary fiber fermentation in the cecum and colon by gut bacteria. Acetate can be generated by *Akkermansia muciniphila*, *Bacteroides* spp., *Blautia hydrogenotrophica*, *Clostridium* spp., *Bifidobacterium* spp., *Prevotella* spp., *Ruminococcus* spp.,and *Streptococcus* spp.^10^, ^11^, ^12^ Buyrate is produced by *Anaerostipes* spp., *Coprococcus comes*, *Clostridiales bacterium, Coprococcus eutactus, Coprococcus catus*, *Costridium symbiosum, Eubacterium rectale*, *Eubacterium hallii*, *Faecalibacterium prausnitzii*, *Roseburia insulinivorans* and *Roseburia intestinalis*.^11^^,^^13^^,^^14^ Propionate is also generated through *Akkermansia muciniphila*, *Bacteroides* spp., *Clostridium* spp*., Coprococcus* spp*., Dialister* spp., *Eubacterium* spp., *Phascolarctobacterium succinatutens*, *Roseburia* spp.*, Ruminucocus* spp., and *Veillonella* spp.^11^^,^^13^^,^^15^

### BA Derivatives

Two primary BAs, cholic acid (CA) and chenodeoxycholic acid (CDCA), are generated in the liver. These primary BAs are then conjugated, deconjugated, and transformed into other derivatives in gut microbiota. Bacteria such as *Actinobacteria hathewayi*, *Bacteroides vulgatus*, and *Lactobcillus (L) ruminis* can conjugate glycine to deoxycholic acid (DCA), CDCA, or CA *in vitro*^16^ Bacteria *Clostridium scindens*, *Bacterorides vulgatus*, *L. ruminis,* and *Holdemania filiformis* can also conjugate CDCA, DCA, or CA to one or more amino acids such as alanine, arginine, and aspartate.^16^ Notably, 4 distinct ways are used to transform BAs, including deconjugation, dehydroxylation, oxidation, and epimerization in human.^17^ Conjugated BAs may be deconjugated in gut bacteria^18^ such as *Bifidobacterium* spp.,^19^ *Bacteroides* spp.,^20^ *Clostridium* spp.^21^^,^^22^ and *Enterococcus* spp.,^23^ and *L.* spp.^24^ While BAs are deconjugated, BAs can be converted into secondary BAs, ie, DCA and lithocholic acid (LCA) through bacterial BA hydrolases^25^ and dehydroxylases^26^ to remove 7α or 7β-hydroxyl groups from primary BAs in the large bowel^27^ A range of oxo-, epi- and iso-derivatives such as 3-oxoLCA, 7-oxoCDCA, 12-oxoCA, 7-oxoCA, 12-oxoDCA, iso-LCA, 3-oxo-LCA, 3-oxoallo-LCA, isoalloLCA, allo-LCA, and 3-ketoLCA are also found in gut bacteria^17^^,^^28^^,^^29^ such as *Adlercreutzia*, *Bifidobacterium*, *Clostridium*, *Collinsella*, *Enterocloster*, *Eggerthella*, *Gordonibacter*, *Peptoniphilus*, *Phocea*, *Monoglobus*, *Raoultibacter*, and *Mediterraneibacter*, and also *Clostridium absonum, Collinsella aerofaciens, Stenotrophomonas maltophilia,* and *Ruminococcus gnavus*^30^^,^^31^ 7α-epimerization to ursodeoxycholic acid (UDCA) also occurs in *Clostridium baratii*^17^ However, the exact gut bacterium species to produce these BA metabolites need to be further identified.

### Trp Metabolites

Trp metabolites are generated by gut bacteria such as indole by *Bacteroides ovatus*, *Clostridium limosum*, *Enterococcus faecalis,* and *E. coli*^32^; Indole-3-acid-acetic (IAA) and indole-3-propionic acid (IPA) by *Bifidobacterium* spp. and *Clostridium bartlettii*, and *Clostridium sporogenes*^33^, ^34^, ^35^; Indoleacrylic acid and IPA by *Peptostreptococcus* spp^36^; Indole-3-aldehyde by *L. johnsonii*, *L. reuteri, L. acidophilus,* and *L. murinus*^37^; Skatole by *Bacteroides* spp. and *Clostridium* spp^35^^,^^38^; and tryptamine by *Clostridium sporogenes* and *Ruminococcus gnavus* [50]. In addition, *Bacillus, Burkholderia*, *Pseudomonas*, *Stenotrophomonas*, *Xanthomonas,* and *Shewanella* also encode enzymes homologous to those of the eukaryotic kynurenine (Kyn) pathway to produce Kyn and downstream metabolites such as 3-hydroxyanthranilic acid (3-HAA).^39^

In addition, inosine is a purine metabolite of *Akkermansia muciniphila* and *Bifidobacetrium pseudolongum*.^40^ It is also produced by *Bacillus subtilis*, *Corynebacterium ammoniagenes*, and *E. coli*. Hydrogen sulfide (H_2_S) is generated by sulfur-metabolizing bacteria such as *Desulfovibrio* spp. These bacteria can metabolize taurine (*Bilophila wadsworthia*) or cysteine (*Fusobacterium nucleatum*) to form H_2_S.^41^

## The Roles of GMMs in CRCs

**Figure 1:**
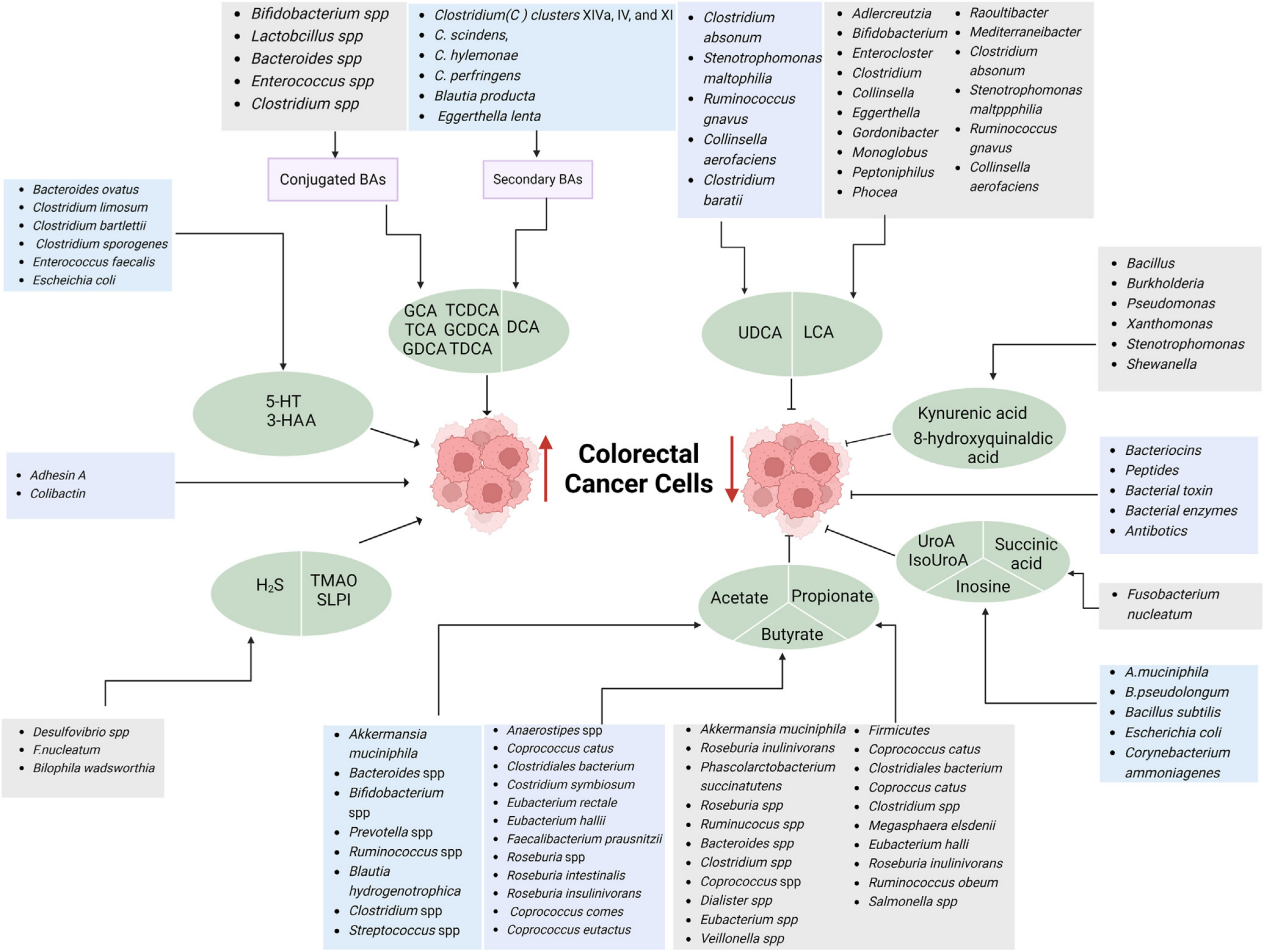
Suppression or promotion on CRCs by GMMs. BAs such as GCA, TCDCA, TCA, GCDCA, GCDCA and TDCA, and secondary BAs such as DCA generated by bacteria can promote colorectal cancer growth; 5-HT and 3-HAA from bacteria also promote colorectal cancer growth; Colorectal cancer growth can be promoted by H2S, TMAO, SLPI, and bacteria toxins such as adhesin A and colibactin derived from bacteria. Notably, GMMs such as UDCA, LCA, kynurenic acid, 8-hydroxyquinaldic acid, UroA, IsoUroA, succinic acid, inosine, acetate, propionate and butyrate generated by bacteria can also inhibit colorectal cancer. 3-HAA, 3-hydroxyanthranilic acid; 5-HT, 5-hydroxytryptamine; BAs, bile acids; CRC, colorectal cancer; DCA, deoxycholic acid; GCA, glycocholic acid; GCDCA, glycoanthropodeoxycholic acid; GCDCA, glycodeoxycholic acid; GMM, gut microbiota metabolite; H2S, hydrogen sulfide; LCA, lithocholic acid; SLPI, secretory leukocyte protease inhibitor; TCA, taurine-conjugated cholic acid; TCDCA, taurochenodeoxycholic acid; TDCA, taurodeoxycholic acid; TMAO, trimethylamine N-Oxide; UDCA, ursodeoxycholate.

GMMs such as SCFAs, BA, and Trp metabolites from bacteria not only directly inhibit but also promote the occurrence and development of CRCs (Figure 1).

### Inhibition on CRCS by GMMs

Gut microbiota-derived peptides, bacterial toxin, bacteriocins, bacterial enzymes, and antibiotics are directly against cancer. However, recent studies found that SCFAs, BA, and Trp metabolites from gut microbiota can also inhibit CRCs.

#### SCFAs

Studies have shown that lower fecal concentrations of 3 major SCFAs (acetate, butyrate, and propionate) are associated with higher risk of CRCs and incidence of CRCs. A significantly lower abundance of SCFAs and SCFA-producing bacteria has also been demonstrated in the CRCs. These SCFAs may enter colonic intestinal cells through passive diffusion, counter-transport with bicarbonate, monocarboxylic acid transporter 1, and sodium-coupled monocarboxylic acid transporter-1.^42^ They have exhibited protective actions against colon carcinogenesis. The inhibitory mechanism shows a close association with the inhibition of histone deacetylases (HDACs). The transformation of glycolytic metabolism is a dominant characteristic of carcinoma cells.^43^ Colorectal cells prefer glucose to butyrate as their preferred energy resource due to Warburg effect pathway. Butyrate not only can enter the nucleus directly to inhibit HDAC but also cause a reduction in short-chain acyl-CoA dehydrogenase levels, which are the primary process in the catalyzation of mitochondrial butyrate oxidation.^44^ This process reduces the auto-oxidation of butyrate in CRC cells to cause accumulation of butyrate in carcinoma cells, thereby restraining CRC development.^45^ SCFAs also inhibit the Wnt/β-catenin signaling pathway by inhibiting HDAC activity, thereby preventing the development of intestinal tumors in a murine model.^46^ Butyrate as a HDAC inhibitor also participates in the apoptosis of CRC cells.^47^ Notably, butyrate can protect from intestinal tumor growth by blocking the activation of calcineurin and nuclear factor of activated T cells C3 in mouse and human.^48^ Interestingly, sodium butyrate could induce ferroptosis in CRC cells through cluster of differentiation (CD)44/secondary lymphoid tissue chemokine 7A11 signaling pathway.^49^ Tributyrin alleviated gut microbiota dysbiosis to repair intestinal damage in antibiotic-treated mice.^50^ Acetate also inhibited CRC cell multiplication and triggered CRC cell apoptosis in a dose-dependent manner. These effects of acetate were mediated via suppression of the phosphatidylinositol-3-kinase/protein kinase B (PI3K/protein kinase B) pathway.^51^

#### BA metabolites

The UDCA, a secondary BA, which has a chemical structure with DCA but unlike DCA, has been demonstrated to inhibit CRC occurrence. While patients with CRCs take UDCA for a long period of time, these patients are less likely to relapse following the removal of the colorectal adenoma. Clinical metagenomic and metabolomic studies have also revealed links between microbial BA metabolism and inflammatory bowal disease and CRC progression.^52^ Serum concentrations of BAs, particularly downstream microbial metabolites of CA, were strongly associated with increased risk of CRC among women.^52^ Notably, decreasing concentration of hydrophobic BA and increasing hydrophilicity of the bile pool were involved in the suppression of UDCA on CRCs.^53^^,^^54^ UDCA also protected against malignant progression of CRC through TGR5–yes-associated protein.^55^ In addition, LCA had antiproliferative action on different cancer cell lines.^56^

#### Trp metabolites

Alteration of fecal Trp metabolism mediated by microbiota is involved in the pathogenesis of CRCs. Indeed, higher levels of plasma Trp were associated with lower CRC risk^57^; whereas increased serotonin, a metabolite of Trp, was related to a higher risk of CRCs. The Kyn-to-Trp ratio was also positively associated with CRCs.^58^ Supplementation with *L. reuteri*, which produces Trp metabolites, inhibits SREBP2 expression and tumorigenesis in mice with gut flora disequilibrium. In addition, kynurenic acid, a Trp metabolite, could inhibit proliferation of several cancer cell lines including CRCs.^59^ 8-hydroxyquinaldic acid, another Trp metabolite inhibited migration of CRC cell lines colorectal cancer cell line and LS-180 and increased the expression of β-catenin and E-cadherin. The Trp metabolism also was effective in inducing the differentiation of Tregs.

#### Others

Other GMMs are also associated with tumor growth such as that inosine, a purine metabolite of *A. muciniphila* and *B. pseudolongum* is effective in controlling cell growth and tumor evolution.^60^ Ellagitannins and ellagic acid are dietary polyphenols, which are poorly absorbed but extensively metabolized by the human gut microbiota to produce different urolithins (Uros). Produced UroA and isoUroA can induce apoptosis in colorectal tumor cell line caco-2 cells through arresting in S and G2/M phases of cell cycle. Kasimsetty et al^61^ also confirm the time- and dose-dependent anticlonogenic and proliferative activity of UroA in human CRC cells (HT-29) through cell-cycle arrest in the G0/G1 and G2/M stages and apoptosis induction. In addition, F. nucleatum-derived succinic acid also induced tumor resistance to immunotherapy in CRCs.

### Promotion on CRCs by GMMs

There is a matter of debate in a causal relationship between gut microbiota and colorectal tumorigenesis. However, studies have shown that the dysbiosis of gut microbiota is related to CRCs. Specific gut bacteria, such as *Fusobacterium nucleatum*, *Escherichia coli,* and *Bacteroides fragilis* could be involved in colorectal carcinogenesis. These bacteria produce metabolites, such as H_2_S, trimethylamine-N-oxide (TMAO), which are likely to promote inflammation and subsequently cancer development.^62^ Previous reports also showed that bacterial toxins such as toxin from *B. fragilis*, adhesin A from *F. nucleatum* and colibactin from *E. coli*^6^^,^^63^ can promote the occurrence and development of CRCs. They participate in metabolism of dietary components to produce tumorigenic metabolites, interaction with genetic or epigenetic alterations, induction of inflammation and the recruitment of immunosuppressive cells. Indeed, colibactin could cause the breakage of double-strand DNA, chromosome instability, and cell senescence in eukaryotic cells.^64^ Notably, the gut microbiota also interacted with certain environmental factors such as a high-fat diet (HFD)^65^ and cigarette smoking^66^ to promote the development of CRCs. The fat-mediated alterations of the gut microbiota linked BA metabolism to CRC risk and colonic tumorigenesis.^67^ Cigarette smoke also promoted CRC through modulation of gut microbiota and related metabolites.^66^ However, recent studies have also found that GMMs such as secondary BAs and Trp metabolites are related to the promotion on CRCs.

#### BA metabolites

The secondary BAs have exhibited procarcinogenic properties. Paired microbiome and metabolome analyses were associated with BA changes with CRC progression. The plasma levels of 7 conjugated BA metabolites, including glycocholic acid, taurine-conjugated CA, glycodeoxycholic acid, taurochenodeoxycholic acid, and taurodeoxycholic acid (TDCA) were positively correlated with risk of colon cancer. Metabolomic analysis showed increased BA metabolite TDCA in the colon of smoke-exposed mice^66^ and in the colon of individuals consuming HFDs, which were associated with an increased risk of CRCs. HFDs also promoted colorectal tumorigenesis through modulating gut microbiota and metabolites.^65^ HFD feeding could increase CRC growth in β2AR-dependent manner.^68^ Interestingly, germ-free mice transplanted with stools from smoke-exposed mice had increased colonocyte proliferation.^66^ Cigarette smoke could promote CRC through modulation of gut microbiota and related metabolites.^66^ The secondary BAs fed mice also increased inflammation and induced CRCs.^69^ Studies found that hydrophobic BAs can produce reactive oxygen species (ROS) and reactive nitrogen substances, which cause oxidative stress, damage to DNA and proteins, and destruction of the base excision repair pathway. In the carcinogenic methane peroxide-induced tumor model system, DCA raises the rate of CRCs in Kirsten rat sarcoma viral oncogene homolog point mutual mutations. DCA can also enhance the local spatial aggregation of phospholipid acid and induces co-localization between phospholipid acid and epidermal growth factor receptor (EGFR) to promote EGFR dimerization/oligomerization and EGFR-MARK signaling. EGFR, as a tyrosine kinase receptor can promote proliferation, invasion or metastasis of CRCs by mutation or overexpression. Notably, unconjugated BAs and tertiary BAs are not associated with cancer risk.

#### Trp metabolites

Trp metabolites serotonin (5-HT) and 3-hydroxyanthranilic acid (3-HAA) can facilitate tumor cells such as CRCs to escape from ferroptosis. Mechanistically, both 5-HT and 3-HAA were as potent radical trapping antioxidants to eliminate lipid peroxidation, thereby suppressing ferroptotic cell death.^70^ During this process, monoamine oxidase A markedly abrogated the protective effect of 5-HT via degrading 5-HT. Kynureninase, which was essential for 3-HAA production to confer cells resistant to ferroptotic cell death, whereas 3-hydroxyanthranilate 3,4-dioxygenase significantly blocked 3-HAA mediated ferroptosis inhibition.^70^ 3-IAA, a Trp metabolite can induce a downregulation of the ROS-degrading enzymes glutathione peroxidase 3 and glutathione peroxidase 7 to reduce tumor proliferation.^71^ Probiotic *L. reuteri* promotes interferon-γ-producing CD8 T cells, thereby bolstering immune checkpoint inhibitor (ICI) via its released dietary Trp catabolite I3A.^72^

#### Others

TMAO engaged in a number of genetic pathways has an apparent association to carcinomas, particularly colon cancer. Mechanistically, TMAO can potentially cause CRC by inflammation, DNA damage, oxidative stress, and protein misfolding.^73^ However, it remains uncertain whether elevated TMAO levels are a reason or a result of cancer. The level of H_2_S in the feces of CRC subjects is higher than in the control group without tumors.^74^ H_2_S promotes the proliferation of CRCs via promoting the phosphorylation of Please add expansions for protein kinase B, secondary lymphoid tissue chemokine, CD, inflammatory bowal disease, TGR, HT, Kirsten rat sarcoma viral oncogene homolog, extracellular regulated protein kinase, interluekine, G protein-coupled bile acid receptor 1 (GPBAR1), GATA, PD-L, CRCS and extracellular regulated protein kinase *in vitro*. In addition, microbiota-derived genotoxin tilimycin also caused colonic stem cell mutations. Urea cycle activation triggered by host-microbiota maladaptation could drive colorectal tumorigenesis. Dietary iron also modulated gut microbiota and induced secretory leukocyte protese inhibitor secretion to promote colorectal tumorigenesis.

## Effects of GMMs Associated Immune Cells on the CRCs

**Figure 2:**
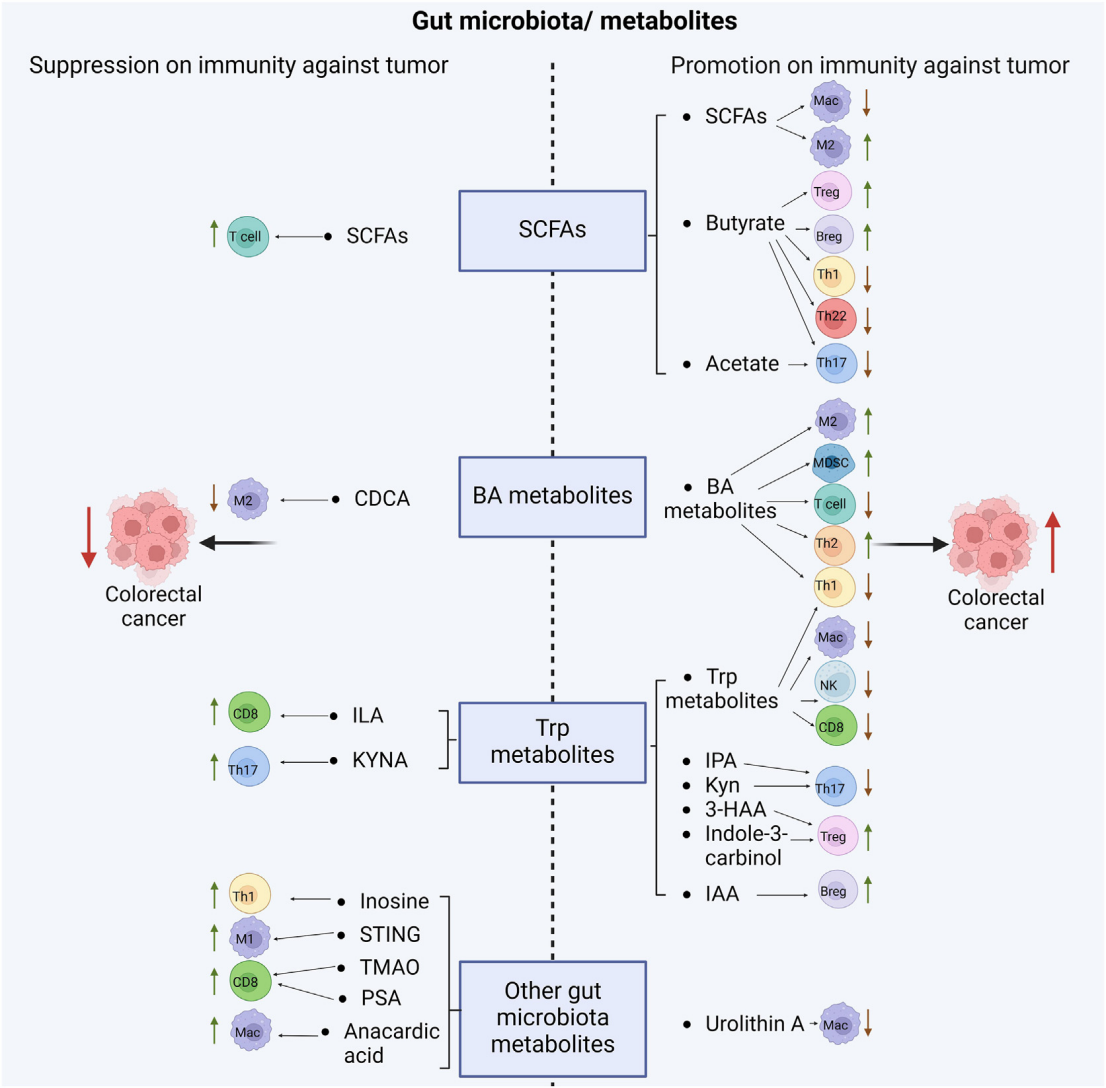
Suppression or promotion on CRCs by GMM-associated immune cells. SCFAs promote colorectal cancer growth through downregulating Mac and upregulating M2. Notably, SCFA butyrate promotes colorectal cancer through upregulating Treg and Breg, and downregulating Th1, Th22 and Th17; Colorectal cancer is also promoted through acetate downregulating Th17; However, SCFAs also inhibit colorectal cancer through T cells. BA metabolites promote colorectal cancer through upregulating M2 and MDSC and downregulating Th2 and Th1; Whereas BA metabolite CDCA also inhibits colorectal cancer through downregulating M2. Trp metabolites promote tumor growth through downregulating Th1, Mac, NK and CD8. Notably, Trp metabolites IPA and Kyn, 3-HAA and indole-3-carbinol as well as IAA promote tumor growth through downregulating Th17 and upregulating Treg and Breg respectively; However, Trp metabolites ILA and KYNA also inhibit tumor through upregulating CD8 and Th17; Other GMM such as Urolithin A promote colorectal cancer; Whereas inosine, STING, TMAO, PSA and anacardic acid can inhibit colorectal cancer through upregulating Th1, M1, and CD8. 3-HAA, 3-hydroxyanthranilic acid; 3-oxoLCA, 3-oxo-lithocholic acid; 5-HIAA, 5-hydroxyindole-3-acetic acid; BA, bile acid; Breg, regulatory B cells; CDCA, chenodeoxycholic acid; CRC, colorectal cancer; GMM, gut microbiota metabolite; IAA, indole-3-acetic acid; ILA, indole-3-lactic acid; IPA, indole-3-propionic acid; IsoalloLCA, isoallo-lithocholic acid; KYN, kynurenine; KYNA, kynurenic acid; L. plantarum, *Lactobcillus* plantarum; LCA-3-S, sulfate of LCA; M1, M1 macrophages; M2, M2 macrophages; Mac., macrophages; MDSC, myeloid-derived suppressive cells; NK, natural killer cells; PSA, polysaccharide; SCFAs, short chain fatty acids; Th, T helper; Treg, regulatory T cells; Tr1, T regulatory cells; Trp, tryptophan; THDCA, taurohyodeoxycholic acid; STING, stimulator of interferon genes; TMAO, trimethylamine N-Oxide.

GMMs play a critical role in maintaining homeostasis of gut and systemic immunity; however, they also promote immune responses against CRCs (Figure 2). Within CRC microenvironment, there are multiple antitumor immune cells, including multiple kinds of immune cells such as effective CD8 cells, CD4 Th1 cells, Th17 cells, natural killer (NK) cells, dendritic cells (DCs) and inflammatory Macs; whereas other immune cells such as MDSCs, immunosuppressive Macs (M2-like Macs) and N2 neutrophils, Tregs and Bregs can contribute to tumor growth. These tumor-associated immune cells can be regulated by GMMs. Notably, these GMM-mediated immune cells also produce effects on the tumor environments such as that Treg cells in tumor environment can produce vascular endothelial growth factor (VEGF) to promote angiogenesis, and inhibit T effective (Teff) cells, NK cells and antigen-presenting cells by inhibitory cytokines such as IL-10 and cytotoxic molecules such as granzymes and perforin, which can directly kill Teff cells and antigen-presenting cells.^75^

### Suppression on Immunity Against CRCs by GMMs

#### SCFAs

SCFAs have an important role in maintaining immune homeostasis through their receptors such as G-protein coupled receptor (GPR)43. Nucleotide oligomerization domain–like receptor thermal protein domain associated protein 3 (nucleotide oligomerization domain–like receptor 3 (NLRP3))–mediated inflammatory signaling pathways could be negatively regulated by SCFAs to inhibit the activation of Macs. SCFAs also triggered autophagy in cancer cells to promote M2 polarization in Macs, accelerating tumor advancement. Notably, under Treg cell polarization condition, SCFAs could promote the conversion of naïve T cells toward Tregs.^76^, ^77^, ^78^ SCFA butyrate also suppressed Th17-associated retinoid-related orphan receptor γt (RORγt) and increased the expression of Treg-associated Foxp3.^79^ DCs exposed to butyrate facilitated the differentiation of naïve T cells into Foxp3^+^Tregs.^80^ Administration of SCFAs also increased the Bregs (B regulatory cells) frequency.^81^ In addition, gut microbiota SCFAs also affected immune effective cells such as that butyrate could decrease the proliferation and cytokine production in Th1, Th17, and Th22 cells.^82^

#### BA metabolites

BA metabolites have widely effects on immune cells through receptors such as TGR5 (GPBAR1), farnesol-X-receptor, vitamin D receptor (VDR), liver X receptor, pregnane X receptor (PXR), and RORγt. They are essential to maintain tolerant phenotypes of the Macs via TGR5 (GPBAR1). Secondary BAs DCA and LCA could act as endogenous inhibitors of NLRP3 activation by activating TGR5^83^ or TGR5-cAMP(adenosine monophosphate)-dependent ubiquitination of NLRP3^84^ farnesol-X-receptor, another BA receptor, is an important negative regulator of NLRP3.^84^ BA metabolites could also disrupt intracellular calcium homeostasis, which is essential for nuclear factor of activated T cells signaling and T cells activation.^85^ 24- NorUDCA, a BA metabolite, reshaped immunometabolism in CD8^+^ T cells,^86^ which affected lympho-blastogenesis, expansion, glycolysis and target of rapamycin complex 1 (mTORC1) signaling. Notably, the physiological concentrations of unconjugated LCA could inhibit the activation of primary human and mouse CD4^+^ Th1 cells through a VDR-dependent mechanism, resulting in decreased tumor necrosis factor-α and IFN-γ.^87^ VDR activation also promoted a shift from Th1 to Th2 phenotype through increased production of the transcription factors c-Maf and GATA-3.^88^ Increased number of tumor-infiltrating Treg cells is associated with poor prognosis in various cancer types.^89^ Notably, BA metabolites such as 3-oxoLCA, isoalloLCA, and isoDCA have been identified as the regulators of Treg cells by inhibiting the differentiation of Th17 cells and increasing the differentiation of Treg cells in colon. NR4A1 (Nuclear receptor subfamily 4 group A member 1) was also required for the role of isoalloLCA in Treg cells.^90^ IsoalloLCA could increase binding of NR4A1 at the Foxp3 locus to enhance Foxp3 gene transcription. The differentiation of Th17 cells was also inhibited by 3-oxoLCA by RORγt,^91^ which finally affected the Th17/Treg balance. The sulfate of LCA (LCA-3-S) selectively suppressed Th17 cell differentiation without influence on Th1, Th2, and Treg cells.^92^ Taurohyodeoxycholic acid, a natural 6α-hydroxylated BA, not only reduced the secretion of Th1/Th17-related cytokines and transcription factors, but also increased the production of Th2/Treg-related cytokines and the expressions of transcription factors in the colon.^93^ In addition, the BA derivative TDCA also increased MDSCs in the spleen of septic mice.^94^ MDSCs, as immunosuppressive cells antagonized the activities of T cells by depleting amino acids, and expressing transforming growth factor beta (TGFβ) and PD-L1.^95^ Notably, intratumoral enrichment of *F. nucleatum* in patients with CRC was associated with enrichment of MDSCs.^96^ Taurodeoxycholic acid (TDCA) also increased the number of MDSCs in the spleen of septic mice.^94^

#### Trp metabolites

Trp metabolites have an important role in the differentiation and function of immune regulatory cells through their receptors such as ary hydrocarbon receptor (AHR). Administration of AHR ligand indole-3-carbinol, a Trp metabolite, was sufficient to restore Treg compartment.^97^ Notably, *in vitro* experiments also verified that Trp metabolites IPA inhibited the differentiation of Th17 cells and promoted the differentiation of Treg cells.^98^ Notably, during T regulatory 1 (Tr1) cell differentiation, AHR could be physically associated with c-Maf to activate immunosuppressive cytokines IL-10 and IL-21 promoters.^99^^,^^100^ Differentiation and function of IL-10-producing CD19^+^CD21^hi^CD24^hi^Bregs were also regulated by AHR.^101^ Trp metabolite IAA from gut microbiota together with LPS could activate transcription factor PXR and nuclear factor-κB to induce the generation of IL-35^+^ Breg cells.^102^ In addition, Kyn from gut microbiota could enhance differentiation of Tregs through the activation of AHR.^103^, ^104^, ^105^, ^106^, ^107^, ^108^ 3-HAA, a downstream metabolite of Kyn promoted the generation of Foxp3^+^Treg cells and immunosuppressive TGF-β in a nuclear coactivator 7 (NCOA7)-dependent pathway.^109^ Microbiota-derived Trp catabolites mediated the chemopreventive effects of statins on CRC by inhibiting Th 17 cell differentiation by targeting the nuclear receptor, retinoic acid receptor-related orphan receptor γt (RORγt).^110^ Notably, Trp metabolites could also activate AHR in tumor-associated Macs to suppress antitumor immunity^111^ such as that the metabolites of dietary Trp generated by the gut microbiota activated the AHR in myeloid cells, promoting an immune suppressive tumor microenvironment.^112^ In addition, Trp metabolites were also involved in the regulation of immune effective cells. programmed cell death protein-1 (PD-1), an immunosuppressive molecule in CD8^+^T cells could be upregulated through AHR.^113^ 3-HAA metabolite of Kyn could induce apoptosis of T-cells.^114^ L-kyn metabolites also caused NK cell death via ROS pathway.^115^

#### Others

Gut microbiota urolithin A alleviated colitis in mice by improving gut microbiota dysbiosis, modulating microbial Trp metabolism, and triggering AHR activation.^116^ Urea from *Bifidobacterium*, could enter into Macs to inhibit the binding efficiency of p-signal transucer and activator of transcription 1 to promotor region, and further skew Macs toward a protumor phenotype characterized by the accumulation of polyamines.^117^

### Promotion on Immunity Against CRCs by GMMs

#### SCFAs

There are contradictions about the effects of SCFAs on the immune cells. Besides, SCFAs play an important role in maintaining homeostasis, SCFAs also increase tumor-killing CD8^+^ T cells and reduce immune-suppressing Tregs in tumor tissues such as that the supplements using SCFAs or SCFA-producing bacteria increase intratumor T cells and raise the concentration of cytokines interferon-γ and tumor necrosis factor-α to result in inhibition of tumor growth.^118^ Decreased abundance of SCFA-producing taxa such as *Coprococcus* was subsequently associated with a lower number of intratumoral CD4^+^ and CD8^+^cells and peripheral CD4^+^ T cells. He et al demonstrated that butyrate could promote the antitumor activity of intratumoral and draining lymph node CD8^+^ T cells in an IL-12 signaling pathway-dependent manner in a mouse model.^119^ Thus, SCFAs have beneficial effects in CRCs.

#### BA metabolites

BA metabolite CDCA suppressed M2 Mac polarization.^120^ Mechanistically, it could cause mitochondrial morphology damage, including swelling and reduction of cristae, decreased mitochondrial membrane potential, and elevated mitochondrial calcium level, which resulted in the production of ROS.

#### Trp metabolites

*L. plantarum*–derived indole-3-lactic acid, a Trp metabolite, could ameliorate colorectal tumorigenesis via epigenetic regulation of CD8^+^ T cell immunity. Interventions with *L. reuteri* or its metabolite indole-3-lactic acid could complement chemoprevention strategies for CRCs.^110^ Kynurenic acid also modulated the recruitment and aggregation of GPR35-positive Macs to cause a robust Th17 immune response.

#### Others

Both *A. muciniphila* and *L. rhamnosus* activated stimulator of interferon genes–interferon pathways to slow tumor progression. *Bifidobacterium*, another kind of bacteria, altered the functional capacity of DCs to induce CD8^+^T cell proliferation and IFNγ production.^121^^,^^122^ It also promoted Th1 transcriptional differentiation and antitumor immune responses to improve immunocheckpoint blockage efficacy.^40^ *Bacteroides fragilis* induced Mac polarization to M1 and upregulated CD80 and CD86 expression on the cells, which could promote innate immunity.^123^ *Enterococcus hirae* was able to induce the polarization of immune cells in secondary lymphoid organs towards a Th1 IFNγ phenotype, leading to increased ratios of cytotoxic T cells to Tregs in mouse models. *Eleven* strains combined with immunocheckpoint blockages robustly induced IFN γ^+^CD8^+^T cells to inhibit tumor growth. *Faecalibacterium* increased CD4^+^T cell proportion and also reduced Treg cell proportion in peripheral blood. *L. plantarum* effectively increased expression of the natural cytotoxic receptors, and promoted NK cell activation to trigger innate immunity.

## Effects of GMMs on the Formation of CRC Metastasis

### Potential Effects of GMM-Mediated Tumor-Associated Macs (TAMs) and MDSCs on CRC Metastasis

**Figure 3:**
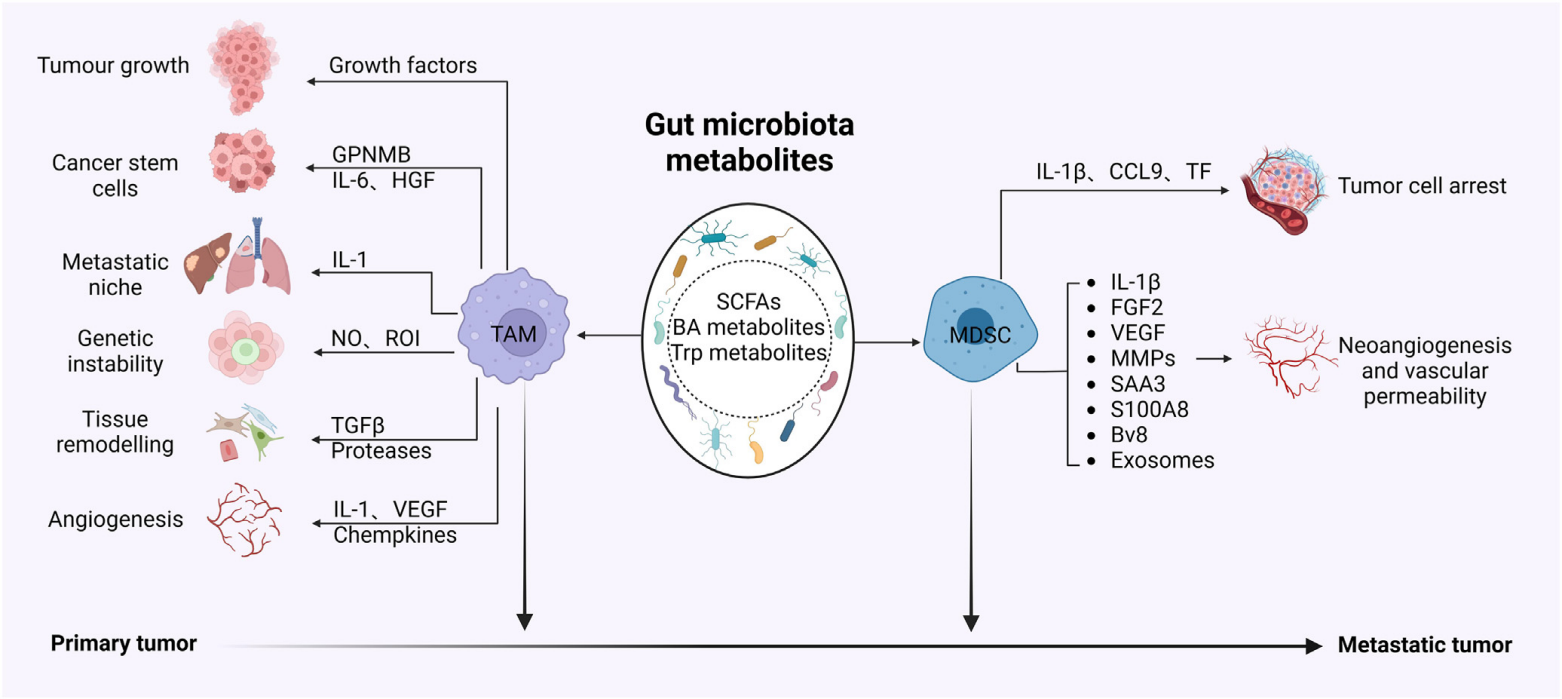
Effects of GMMs on CRC metastasis via Macs and MDSCs. TAMs and MDSCs promote the formation of metastatic tumor. SCFAs, BA, and Trp metabolites can promote colorectal cancer metastasis through causing tumor cell arrest by MDSC derived IL-1β, CCL9 and TF, and promoting tumor neoangiogenesis and vascular permeability by IL-1β, FGF2, VEGF, MMPs, SAA3, S100A8, Bv8 and exosomes from MDSCs; SCFAs, BA, and Trp metabolites also promote colorectal cancer metastasis through improving tumor growth by growth factors and cancer stem cells by GPNMB, IL-6, and HGF, causing metastatic niche by IL-1 and genetic instability by NO and ROI, leading to tissue remodeling by TGFβ and proteases, and angiogenesis by IL-1, VEGF and chemokines from TAMs. BA, bile acid; CCL9, C-C motif chemokine 9 protein; CRC, colorectal cancer; DCs, dendritic cells; EGF, epidermal growth factor; GMM, gut microbiota metabolite; GPNMB, glycoprotein neuromedin B; HGF, hepatocyte growth factor; MDSC, myeloid-derived suppressive cells; NK, natural killer cells; NO, nitric oxide; ROI, reactive oxygen intermediates; MMP, matrix metalloproteinase; SAA3, serum amyloid A3; SCFAs, short chain fatty acids; Treg, regulatory T cells; TAMs, tumor-associated macrophages; TF, tissue factor; TGFβ,transforming growth factor-β;Trp, tryptophan; VEGF, vascular endothelial growth factor.

There are many factors that regulate the dissemination and distant organ colonization of CRC cells, including genetic mutations, metastasis-initiating cells, epithelial-mesenchymal transition (EMT), and the tumor microenvironment. The GMMs induced immunosuppressive cells such as TAMs and MDSCs can help direct metastatic dissemination by creating a niche which allow tumor colonization. These immunosuppressive cells can achieve protumor functions by generating a proinflammatory cytokines, maintaining an immunosuppressive microenvironment, remodeling the matrix and creating a proangiogenic and proinvasive environment, and secreting growth factors (Figure 3).

For example, GMM-mediated immunosuppressive Macs could release nitric oxide and reactive oxygen intermediates to cause genetic instability during the initiation phase. These Macs also produced epidermal growth factor and mediators such as IL-6, hepatocyte growth factor, and glycoprotein neuromedin B to promote cancer stem cell expansion. These Macs also potentially contributed to metastatic spread by releasing IL-1 and TGFβ, which could be involved in extracellular matrix (ECM) remodel and pathological fibrosis at later stages. In addition, these Macs also are a critical source of angiogenic factors such as VEGF and proangiogenic chemokines.

Gut microbiota associated MDSCs are also indispensable for the formation of pre-metastatic niche, which promote the niche to favor tumor cell colonization and promote metastasis.^124^ Indeed, *Peptostreptococcus anaerobius* induced chronic inflammation and modulated tumor microenvironment by recruiting MDSCs, TANs (Tumor associated neutrophils) and TAMs.^125^ *F. nucleatum* also caused liver metastasis by modulating liver microenvironment with accumulation of MDSCs, and reduction of NK and Th17 cells. Thus, host microbiota metabolites mediated immunosuppressive cells could promote CRC metastasis. Recent studies have shown that tumor-associated neutrophils (TANs), which can be polarized to antitumorigenic N1 and protumorigenic N2 phenotype, can form web-like structures known as neutrophil extracellular traps (NETs) both in primary tumor microenvironment and metastatic sites. Interestingly, NETs were involved in cancer progression and metastasis.^126^ N2 neutrophils support edtumor growth by expressing arginase, metalloproteinase 9 (MMP-9), VEGF, CCL2, CCL5 and CXCL4.^126^

### Direct Roles of GMMs in CRC Metastasis

**Figure 4:**
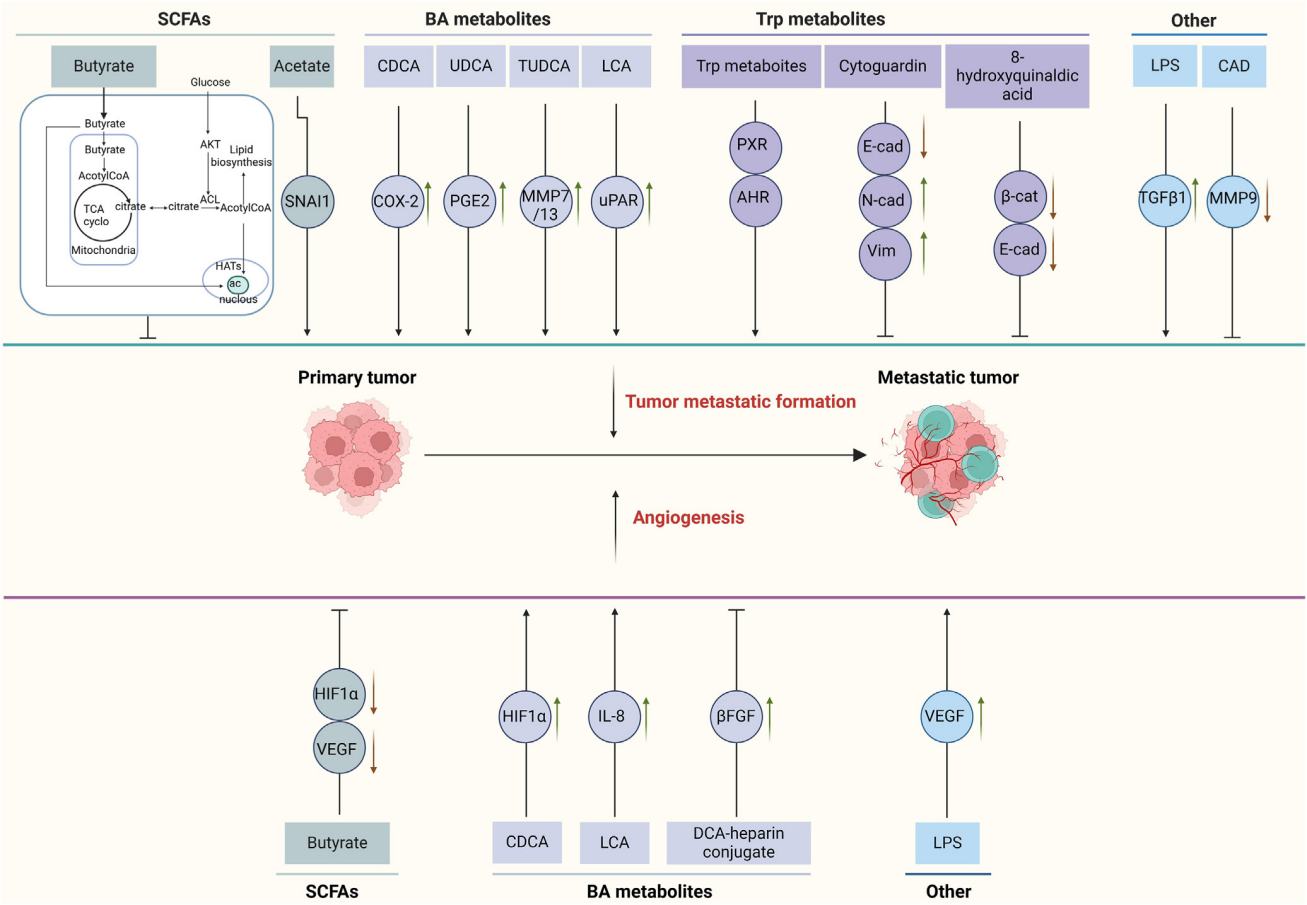
Effects of GMMs on tumor metastasis. Tumor metastatic formation from primary tumor to metastatic tumor includes tumor metastatic formation and angiogenesis. They can be affected by SCFAs, BA and Trp metabolites, and other metabolites. SCFA acetate promotes this process through SNAl1, whereas butyrate inhibits this process through Warburg effect; BA metabolites CDCA, UDCA, TUDCA, and LCA can promote metastasis of tumor through upregulating COX-2, PGE2, MMP7/13 and uPAR respectively. Trp metabolites also promote metastasis of tumor through PXR and AHR, whereas metastasis can be inhibited by cytoguardin through downregulating E-cad and upregulating N-cad and Vim. 8-hydroxyquinaldic acid also inhibits metastasis through downregulating β-cat and E-cad. Other metabolites such as that LPS promotes metastasis through upregulating TGFβ1; Whereas CAD can inhibit metastasis through downregulating MMP9. Angiogenesis play an important role in tumor metastatic formation. SCFAs can inhibit the angiogenesis by downregulating VEGF and HIF1α. Notably, angiogenesis can be promoted by BA metabolites CDCA and LCA through upregulating HIF1α and IL-8 respectively, whereas DCA-heparin conjugate inhibits angiogenesis through upregulating βFGF. LPS also promotes angiogenesis by upregulating VEGF. β-FGF, β fibroblast growth factor; AHR, ary hydrocarbon receptor; BA, bile acid; CAD, cadaverine; COX-2, cyclooxygenase-2; CSC, cancer stem cell; CDCA, chenodeoxycholic acid; DCA, deoxycholic acid; E-cad, E-cadherin; GMM, gut microbiota metabolite; HIF1α, hypoxia-inducible factor 1α; LCA, lithocholic acid; LPS, lipopolysaccharide; MIC, metastasis initiating cell; MMP, matrix metalloproteinase; N-cad, N-cadherin; PGE2, prostaglandin E2; PXR, pregnane X receptor; SNAI1, snail family transcriptional repressor 1; SCFAs, short chain fatty acids; TGFβ, transforming growth factor β; Trp., tryptophan; β-cat,β-catenin; TUDCA, taurousodeoxycholic acid; UDCA, ursodeoxycholic acid; uPAR, urokinase-type plasminogen activator receptor; VEGF, vascular endothelial growth factor; Vim, vimentin.

Tumor metastatic formation from primary tumor to metastatic tumor would pass several stages, including hybrid intermediate cells, transient fetal/regenerative progenitors, differentiated stem cell-like MIC (metastasis initiating cell), dormant MIC and proliferative MIC. Studies have found that some bacteria are involved in the induction of metastasis in primary CRC. They include *Fusobacterium nucleatum*, *Enterococcus faecalis*, *Bacteroides fragilis*, *E. coli* and *Salmonella enterica*. Pathogen *E. coli* upregulated cathepsin K (CTSK) expression which served as a vital mediator between the imbalance of intestinal microbiota and CRC metastasis.^127^ Gut microbiota-stimulated cathepsin K could mediate Toll-like receptor 4 (TLR4)–dependent M2 Mac polarization and promote tumor metastasis in CRC.^127^ Some gut microbiota derived metabolites such as LPS has been found to promote EMT through the upregulation of TGFβ-1 and the activation of nuclear factor-κB.^128^ Clostridium butyricum inhibited EMT of intestinal carcinogenesis through downregulating methyltransferase.^129^ In addition, cadaverine could inhibit EMT in breast cancer cell lines through modulating the expression of MMP-9. However, studies have shown that SCFAs, BA, and Trp metabolites from gut microbiota also directly affect the shaping of tumor metastasis (Figure 4).

#### SCFAs

SCFA butyrate impeded the angiogenesis, metastasis, and survival of CRC cells by inhibiting *Sp1* transactivation via the neuropilin-1/VEGF pathway.^47^ It also reduced clone formation, and migration through the extracellular regulated protein kinase2/mitogen-activated protein kinase pathway. Importantly, butyrate could stimulate the proliferation of normal colonocytes and cancerous colonocytes when the Warburg effect was prevented from occurring.^47^ However, SCFA acetate could promote metastasis by increasing zinc finger protein snail family transcriptional repressor 1 and acyl-CoA synthetase short chain family member 2 in renal carcinoma cells under glucose limitation.^130^

#### BA metabolites

BAs not only participate in the metastatic colonization but also angiogenesis (Figure 4). Differential BA signals emitted by various BA profiles exert distinct pathophysiological traits to the occurrence and development of CRCs. The conjugated BAs promoted CRC-associated liver metastasis. BA metabolite DCA induced EMT and activated VEGF receptor 2.^131^ DCA, as a transcriptional activator in cancer was associated with the enzyme cyclooxygenase 2 in fibroblasts,^132^ which had an influence on tumor microenvironment by increasing the invasiveness and proliferation of cancerous cells. In addition, LCA also contributed to cancer progression and metastasis through inducing the expression of urokinase-type plasminogen activator receptor,^133^ and increasing the expression of matrix MMP genes, including *MMP-1*, *MMP-2*, and *MMP-7.*^134^ Meanwhile, LCA could promote IL-8 expression to stimulate CRC angiogenesis. In addition, taurousodeoxycholic acid, a conjugated form of UDCA, had anti-invasive impacts through decreased expression of MMP-7 and MMP-13 in metastatic breast cancer.^135^ UDCA also decreased the invasiveness of gastric carcinoma cells by interfering with the generation of prostaglandin E2.

#### Trp metabolites

AHR was involved in cancer initiation and metastasis. Trp metabolites, as AHR ligands, participated in the occurrence and development of cancer.^37^ Indeed, D-kyn could promotes epithelial-to-mesenchymal transition via activating AHR.^136^ PXR, another receptor of Trp metabolites, also affected cancer growth, progression, and chemoresistance by regulating the expression of genes implicated in proliferation, metastasis, apoptosis, inflammation, and oxidative stress. Notably, cytoguardin, a Trp metabolite, could resist against cancer growth and metastasis.^137^ 8-hydroxyquinaldic acid, another Trp metabolite, also exerted antiproliferative and antimigratory effects on CRCs.^138^

## Gut Microbiota Elicits CRC-Promoting Inflammation

Inflammation is a risk factor and also a hall marker of CRCs. Colonization with the bacteria such as *F. nucleatum* and *B. fragilis* was associated with colonic inflammation, which was the principal mechanism in promoting colorectal tumorigenesis.^63^^,^^139^ *F. nucleatum* could drive a proinflammatory intestinal microenvironment through metabolite receptor-dependent modulation of IL-17 expression in *Apc*^*min/+*^ mice.^140^ The FadA adhesin/invasin conserved in *F. nucleatum* is a key virulence factor. *B. fragilis* could cause a series of inflammatory reactions due to *B. fragilis* toxin. Others such as TLR2 and TLR4 (TLR2/4) also sense extracellular microbial products, lipopolysaccharide or lipoteichoic acids from Gram-negative and Gram-positive bacteria, activate TLR2/4 signaling to cause downstream expression of proinflammatory cytokines. The nonspecific immunity activation by peptidoglycan involves immunomodulation by different pattern recognition receptors including peptidoglycan recognition proteins, NLRs, TLRs, and C-type lectin receptors. The inflammasomes such as NLRP3 (a central adenosine triphosphatase (ATPase) domain-leucine-rich repeat-pyrin domain-containing proteins 3) also sense pathogen-associated molecular pattern molecules and damage-associated molecular patterns to leads to secretion of IL-1β and IL-18. Recent studies have shown that SCFAs, BA, and Trp metabolites also promote inflammation through immune cells. Indeed, although it has been widely reported that SCFAs play anti-inflammatory roles through different signaling pathways, SCFAs also play proinflammatory roles through G-protein-coupled receptors. For example, Kim et al^141^ found that SCFAs activated GPR41 and GPR43 on intestinal epithelial cells to protect immunity and tissue inflammation in C57BL6 mice without free fatty acid receptor 2 or 3 receptors. The impact of BAs on the inflammatory response was investigated in vitro using Caco-2 cells stimulated by IL-1β. These studies showed that secondary BAs exerted anti-inflammatory effects, but sulfation of secondary BAs abolished their anti-inflammatory properties.^142^ Thus, gut microbiota-elicited inflammation, at least in part, contributes to both the occurrence and development of CRCs.

## Modulation of anti-CRC Immunity by Gut Microbiota

Tumor immunotherapies such as ICIs are effective strategies against tumor. The interactions between gut microbiota and cancer immune responses cause the possibility that gut microbiota can affect tumor immunotherapy. Although there are very few reports on gut microbiota-based methods to enhance immunotherapy efficacy in patients with CRCs, specific bacterial species associated with immunotherapy responses in animal models such as *Bifidobacterium* spp have been found.^143^ Therapeutic methods that target microbiota are being explored, such as fecal microbiota transplantation and probiotics (individual probiotics or cocktails). Studies have found that some species are beneficial responses to immunotherapy. Administration of particular probiotic strains (for instance *Bifidobacterium*) has been revealed to improve the efficiency of immunotherapy via enhancement of PD-1/PD-L1 or cytotoxic T-lymphocyte antigen-4 blockade.^144^ Mager et al^40^ revealed that Bifidobacterium pseudolongum, Lactobacillus johnsonii, and Olsenella species were able to significantly improve the efficacy of ICIs in mouse models of cancer via the production of the metabolite inosine.^40^ B. longum KACC 91563 strain was able to modulate the hosts’ immune system via immunoglobulin production as well as acting via the maintenance and improvement in the Th1/Th2 balance.^145^ Routy et al^146^ observed that the abundance of Akkermansia muciniphila positively affected the clinical responses to ICIs. In addition, prebiotics such as the administration of soluble fibre including inulin and pectin could improve the activity of anti-PD-1 antibodies in various mouse model.^147^ Engineered probiotics and phage-targeted depletion of pathogenic bacteria.^147^^,^^148^ have also been used in therapy against CRC. A tumor-colonizing form of *E. coli* has been engineered to synthesize arginine from ammonia, thus boosting intratumoral L-arginine availability, which is essential for the proper function of cytotoxic T cells.^149^ However, for the development and clinical application of these methods, it is critical to decipher the specialized roles of gut microbiota in regulating the immune responses in CRCs. The findings will give new opportunities to take advantage of our knowledge on the gut microbiota to prevent cancer, augment therapies and reduce adverse effects of treatment.

## Conclusion

We here summarize the effects of GMMs, especially SCFAs, BA, and Trp metabolites on the CRCs and CRC-associated immune cells. SCFAs can inhibit the proliferation of CRC cells, but some of SCFAs also improve immune tolerances to promote development of CRCs. BA metabolites possess carcinogenic nature but some secondary BAs also inhibit the proliferation of CRCs, and importantly induce immune tolerance. Trp metabolites can inhibit CRCs through regulating immune cells. For example, dietary fortification with tributyrin, a butyrate glycerol ester, was shown to arrest the development of CRC in mice, implying that butyrate was an effective anticancer metabolite. UDCA, a secondary BA, produced by *Ruminococcus gnavus* could protect against the development of CRC. However, some of Trp metabolites also directly and indirectly promote the growth of CRCs. Thus, gut microbiota is not only a friend but also a foe of CRCs.

Gut microbiota also directly and indirectly (via immunosuppressive cells) affect tumor metastasis via regulating the shaping of immune-privileged metastatic niches. Since precise and/or personalized treatments of CRCs, which are focused on the gut microbiota, are likely to target either the key cancer-promoting pathogens or the bioactive components or metabolites, all of the findings will increase more opportunities for clinical applications of gut microbiota.
